# Pain and Pain Medication among Older People with Intellectual Disabilities in Comparison with the General Population

**DOI:** 10.3390/healthcare6020067

**Published:** 2018-06-15

**Authors:** Anna Axmon, Gerd Ahlström, Hans Westergren

**Affiliations:** 1 Division of Occupational and Environmental Medicine, Department of Laboratory Medicine, Lund University, SE-221 00 Lund, Sweden; anna.axmon@med.lu.se; 2 Department of Health Sciences, Lund University, SE-221 00 Lund, Sweden; hans.westergren@med.lu.se; 3 Department of Pain rehabilitation, Skane University hospital, 222 85 Lund, Sweden

**Keywords:** cognitive dysfunction, fentanyl, headache, musculoskeletal pain, paracetamol, visceral pain

## Abstract

Little is known about pain and pain treatment among people with intellectual disabilities (IDs). We aimed to describe pain and pain medications among older people with ID compared to the general population. Data on diagnoses and prescriptions were collected from national registers for the period between 2006 and 2012 for 7936 people with an ID and a referent cohort from the general population. IDs were associated with a decreased risk of being diagnosed with headaches, musculoskeletal pain, and pain related to the circulatory and respiratory systems, but they were associated with increased risk of being diagnosed with pain related to the urinary system. Among men, IDs were associated with an increased risk of being diagnosed with visceral pain. People with IDs were more likely to be prescribed paracetamol and fentanyl regardless of the type of pain but were less likely to be prescribed COX(1+2) and COX2 inhibitors and weak opioids. Healthcare staff and caregivers must be made aware of signs of pain among people with IDs who may not be able to communicate it themselves. Further research is needed to investigate whether people with IDs are prescribed paracetamol rather than other pain drugs due to physicians trying to avoid polypharmacy or if there are other reasons not to prescribe a greater range of pain treatments.

## 1. Introduction

The International Association for the Study of Pain (IASP) defines pain as “an unpleasant sensory and emotional experience associated with actual or potential tissue damage or described in terms of such damage” [1,2]. When the pain persists for more than three months [3] and develops into chronicity, several known co-morbidities may occur, such as psychiatric co-morbidity [4,5], sleep disturbance, cognitive and neurological dysfunction, pain sensitization [6], increased disability [7], and an increased risk for falls [8]. The diagnosis and treatment of both acute and chronic pain states are highly dependent of a patient’s ability to communicate both what aggravates and what alleviates their symptoms [9,10,11]. Moreover, chronic pain is a well-known burden for society with large effects on well-being [12]. However, in spite of having been addressed in previous decades, many issues regarding the individual and societal burdens of pain remain unsolved [13]. Moreover, studies have indicated that chronic pain often goes untreated [7,12,14]. Even so, the increase in prescription of strong opioids for chronic pain has added over-use and side-effects on top of the pain problem for many patients [15].

Although the problem of pain among people with intellectual disabilities (IDs) has been identified in some studies [16,17,18], the literature is sparse. However, in clinical settings, it has been reported that pain often goes undetected and undertreated in this group [19,20,21]. As defined by the IASP, pain is an experience that needs to be communicated by the patient to surrounding people in order for the patient to get adequate help, and people with IDs may not be able to provide self-reports of pain [22,23], even when the person possesses verbal skills [24]. Therefore, the identification of pain among them often relies on reports from caregivers. However, caregivers have been found to underestimate the prevalence of pain among people with IDs [20], possibly due to the misconception that the pain threshold among non-communicating people with IDs is high [25].

As in the general population, pain is associated with limitations to daily functioning, emotional well-being, and quality of life among people with IDs [17]. From a societal point of view, pain among people with IDs is a significant factor affecting the number of consultations to general practitioners’ practices [26]. A further problem is that the symptoms of chronic pain may mimic the deterioration of a patients’ general symptomatology, especially regarding psychiatric symptoms or symptoms of cognitive dysfunction [27,28]. Therefore, there are incentives on both individual and financial levels to ensure proper pain management among people with IDs.

In the general population, there is an emerging body of knowledge on pain in the aging population which indicates a general increase in chronic pain for all individuals as they become older [4,29,30,31] and helps to identify a range of treatment options for pain among older people [32,33]. However, even though the life expectancy of people with IDs has increased over recent decades [34] and the number of older people with IDs is increasing rapidly [35], research on pain and pain treatment in this vulnerable group has been scarce. We have previously reported that, although people with IDs have a high prescription rate for a range of drugs, they are less likely than the general population to use prescription drugs for pain management [36]. Moreover, for some drugs, prescription patterns were shown to differ between men and women. However, these data were not linked to information on the diagnosis of pain, and therefore, whether the lower prescription of drugs for pain management was due to a lower prevalence of pain diagnoses remains unknown.

Therefore, the aim of the present study was to describe diagnoses of pain and the prescription patterns of pain medications among older people with IDs in comparison to their age-peers in the general population.

## 2. Materials and Methods

### 2.1. Registers

In Sweden, people with IDs and/or an autism spectrum disorder (ASD) may apply to their municipality for support and service to manage their daily living. These are regulated in the Act Concerning Support and Service for Persons with Certain Functional Impairment (Swedish abbreviation LSS). There are eight different measures of support available for adults: counselling, personal assistance, companion service, contact person, relief service in the home (for informal caregivers), short-time stay away from home (to relieve informal caregivers), special housing, and occupation at daily activities centers. The municipality reports all such support and service to the Swedish National Board of Health and Welfare, and the information is stored in the so called *LSS register*, which was established in 2004. This register contains information about all types of support and services provided. Data are available on the types and amounts of support, the municipality providing the support, and the identification of the person receiving the support. However, although a diagnosis of either ID or ASD is required to receive the support, there is no information regarding any diagnoses recorded in the register.

*The Swedish National Patient Register* contains information on all inpatient and outpatient specialist visits. For each visit, one primary and up to 21 secondary diagnoses are recorded and coded according to the International Statistical Classification of Diseases and Related Health Problems, 10th Revision (ICD-10). The visit is recorded at discharge, i.e., ongoing hospitalizations are not included in the register. Moreover, the register does not cover visits to primary care.

*The Swedish Prescribed Drug Register* was established in July 2005 and contains information on all dispensed prescribed drugs in Sweden, which corresponded to 84% of all drugs sold [37]. Drugs are recorded according to the Anatomic Therapeutic Chemical (ATC) classification system [38]. The ATC system classifies drugs on three levels. The first level comprises a letter and indicates the anatomical main group. For example, drugs with first level “M” are active on the musculoskeletal system. The second level (two digits) indicates the therapeutic subgroup, e.g., muscle relaxant. Information is then added at each level so that the fifth level indicates the chemical substance in the drug.

### 2.2. Study Cohorts

From the LSS register, we obtained information on all people who were at least 55 years old and alive at the end of 2012 and who had received at least one measure of support during that year, regardless of which type of support. We used such support as a proxy for having an ID, and therefore, the 7936 people identified comprised the ID cohort. By using the Swedish Register of the Total Population, Statistics Sweden provided us with a referent cohort (gPop cohort) from the general population, including one-to-one matching by sex and year of birth. Each cohort comprised 3609 (45%) women and 4327 (55%) men. The mean age of participants on 31 December 2012 was 64 years (55–96 years).

### 2.3. Pain

Through the National Patient Register, we collected information for all people in the two study cohorts for the period between 2006 and 2012 and identified visits with at least one diagnosis of pain. Pain diagnoses were categorized as headaches (G43: migraine; G44: other headache syndromes; R51: headache), musculoskeletal pain (M00–M25: arthropathies; M40–M54: dorsopathies; M75: shoulder lesions; M75: enthesopathies of lower limb, excluding the foot; M77: other enthesopathies; M79: other soft tissue disorders, not elsewhere classified), pain related to the circulatory and respiratory systems (R00–R09), visceral pain (pain related to the digestive system and abdomen, R10–R19), and pain related to the urinary system (R30–R39). The National Patient Register contains no information on whether the pain is acute or chronic and we could not, therefore, distinguish between these two types of pain.

### 2.4. Pain Medication

Through the Prescribed Drug Register, we collected information on dispensed drugs for pain treatment between 2006 and 2012. The drug groups considered were COX(1+2) inhibitors (NSAIDs (Nonsteroidal Anti-inflammatory Drugs), M01A) excluding COX2 inhibitors and glucosamine, COX2 inhibitors (M01AH01, M01AH05), paracetamols (N02BE01, N02BE51, N02BE71), strong opioids (morphine (N02AA01, N02AA51, N02AG01), oxycodone (N02AA05, N02AJ17-19), ketobemidone (N02AB01), pethidine (N02AB02), buprenorphine (N02AE01), tapentadol (N02AX06), and fentanyl (N02AB03)), weak opioids (codeine (N02AJ06-09, N02AA59, N02AA79), dextropropoxyphene (N02AC04), and tramadol (N02AX02, N02AJ13, N02AJ15), drugs used for treating migraines except dihydroergotamin (N02CC01-07, N06AX01), antiepileptics used for treating pain (gabapentin (N02AX12), pregabalin (N03AX16), lamotrigine (N03AX09), and topiramate (N03AX11)), tricyclic antidepressants used for treating pain (amitriptyline (N06AA09) and nortriptyline (N06AA10)), and selective serotonin-norepinephrine reuptake inhibitors (SNRIs) used for the treatment of pain (duloxetine (N06AX21) and venlafaxine (N06AX16)). Since fentanyl plaster is used for non-cancer pain in some institutions [39], its use is controversial. As such, we performed separate analyses for fentanyl.

### 2.5. Ethics Approval

Approval was obtained from the Regional Ethical Review Board in Lund (No. 2013/15). The National Board of Health and Welfare and Statistics Sweden performed a separate secrecy review in 2014 before providing access to the data. All analyses were performed using anonymized datasets. The authors assert that all procedures contributing to this work complied with the ethical standards of the relevant national and institutional committees on human experimentation and with the Helsinki Declaration of 1975, which was revised in 2008.

### 2.6. Statistics

Analyses of dichotomous outcomes were performed using generalized linear models (GLM) by estimating relative risks (RRs) with 95% confidence intervals (CIs). For drugs with the main indication being depression (tricyclic antidepressants and SNRIs) or epilepsy (antiepileptics), we performed sensitivity analyses in which all people with at least one diagnosis of depression (F32 and F33 in ICD-10) or epilepsy (G40 and G41), respectively, were excluded.

All analyses were performed using IBM SPSS Statistics version 23.0 (International Business Machines Corporation (IBM), Armonk, NY, USA). Analyses were only performed when both groups were comprised of more than five individuals. A two-sided *p*-value below 0.05 was considered statistically significant.

## 3. Results

### 3.1. Pain

There was a total of 4625 pain diagnoses (whereof 3109 (67%) were primary diagnoses) in the ID cohort and 6294 (4009 (64%) primary) in the gPop cohort. At visits where pain was a secondary diagnosis, the five most common primary diagnoses in the ID cohort were epilepsy (G40), unspecified mental retardation (F79), pneumonia, organism unspecified (J18), fracture of femur (S72), and non-insulin dependent diabetes mellitus (E11). In the gPop cohort, the five most common primary diagnoses were other medical care (Z51), atrial fibrillation and flutter (I48), non-insulin dependent diabetes mellitus (E11), chronic ischemic heart disease (I25), and angina pectoris (I20).

**Figure 1:**
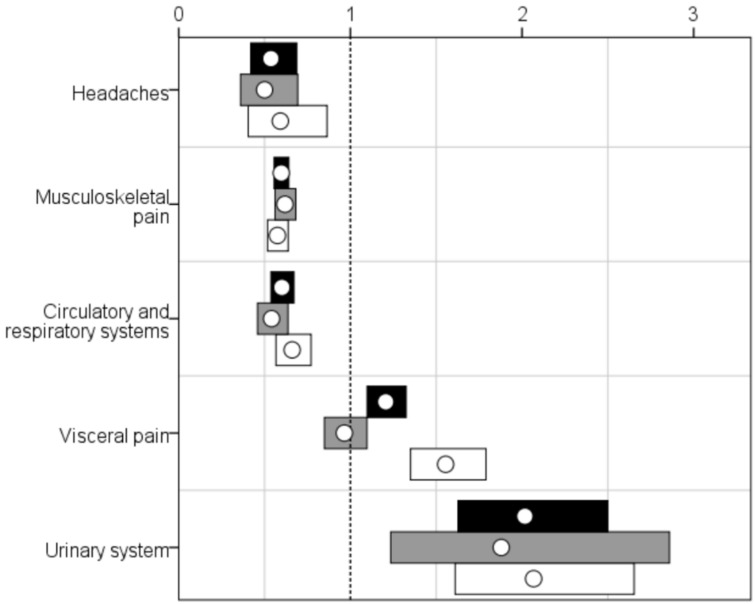
Relative risks (RRs; white dots) with 95% confidence intervals (CIs; bars; black = all, grey = women, white = men) for different pain diagnoses among 7936 older people with intellectual disabilities vs. a random sample from the general population, one-to-one matched by sex and year of birth. Dotted line indicates RR = 1, i.e., CIs not crossing indicate statistically significant RRs.

Musculoskeletal pain was the most common pain diagnosis in both cohorts even though, among people with IDs, visceral pain was also recorded for a large fraction of the cohort (see Table 1). The patterns were similar when stratified by sex. People in the ID cohort were twice as likely as those in the gPop cohort to have had a diagnosis of pain related to the urinary system during the study period (see Figure 1). Among men, but not women, having an ID was associated with an increased risk of having a diagnosis of visceral pain. People with IDs were less likely to have had diagnoses of headache, musculoskeletal pain, or pain related to the circulatory or respiratory system.

### 3.2. Pain Medication

**Figure 2:**
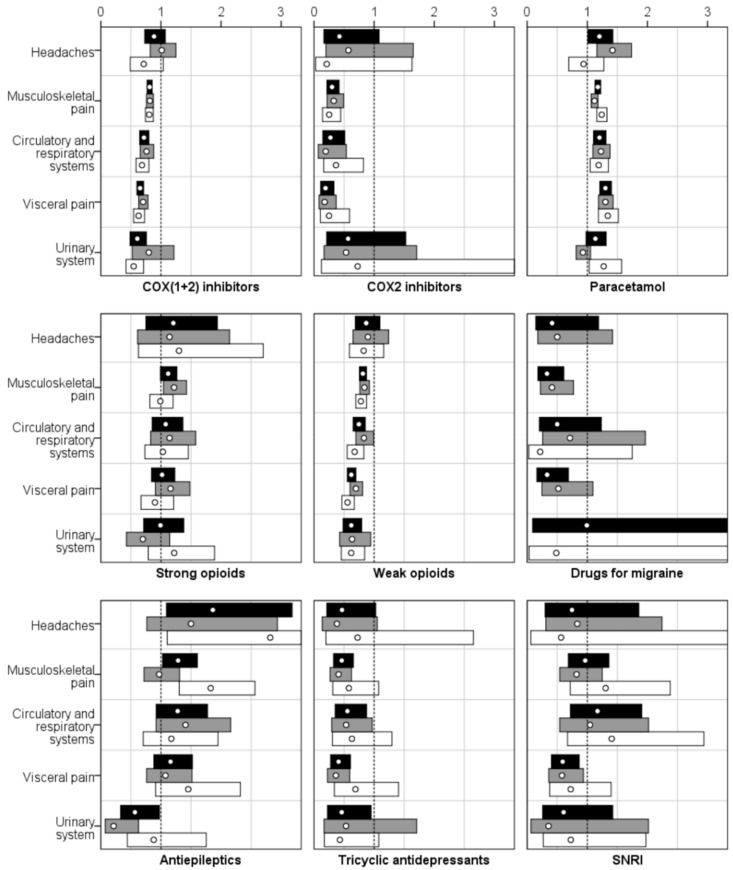
Relative risks (RRs; white dots) with 95% confidence intervals (CIs; bars; black = all, grey = women, white = men) for the prescription of pain medication among 7936 older people with intellectual disabilities vs. a random sample from the general population, one-to-one matched by sex and year of birth, stratified by pain diagnosis. The dotted line indicates RR = 1, i.e., CIs not crossing indicate statistically significant RRs.

People with IDs were more likely than those in the gPop cohort to be prescribed paracetamol for all investigated types of pain diagnoses (see Table 2 and Figure 2). There was, however, a pattern of less prescription of COX(1+2) and COX2 inhibitors as well as for weak opioids associated with ID cohort affiliation.

When excluding those with a diagnosis of epilepsy from the analyses concerning the prescription of antiepileptics, the risk of prescription in the ID cohort decreased (see Table 3). However, the results for prescribing tricyclic antidepressants and SNRIs remained similar when restricting the analyses to those without a diagnosis of depression.

In the ID cohort, 99 people (1.2%) had at least one prescription of fentanyl compared with 47 (0.6%) in the gPop cohort. Among those diagnosed with musculoskeletal pain, 37 people (4%) in the ID cohort and 27 (2%) in the gPop cohort had been prescribed fentanyl (RR 2.30, 95% CI 1.41–3.75). The corresponding numbers for those with pain related to the circulatory and respiratory systems were five (1%) and 15 people (2%; RR 0.56, 0.20–1.52), respectively; for visceral pain, there were 24 (3%) and 13 people (2%; RR 1.53, 0.79–2.99), respectively; and for pain related to the urinary system, there were seven (3%) and five people (4%; RR 0.69, 0.23–2.14), respectively. Less than five people had a prescription of fentanyl and a diagnosis of headaches.

## 4. Discussion

People in the ID cohort were more likely to be diagnosed with visceral pain or pain related to the urinary system. They were less likely to be diagnosed with headaches, musculoskeletal pain, or pain related to the circulatory or respiratory systems. Regardless of the type of pain, people with IDs were more likely to be prescribed paracetamol and fentanyl but were less likely to have a prescription for COX(1+2), COX2 inhibitors, weak opioids, drugs for migraine, or tricyclic antidepressants. Even though no differences were recorded between the groups for strong opioids in general, fentanyl was prescribed to twice as many people in the ID group.

When further interpreting the results from the present study, there are some potential weaknesses that need to be considered. First, using support intended for people with ID or ASD as a proxy for having an ID may have caused a dilution of the ID cohort with people with ASD but without an ID. However, among those with a diagnosis of ID and/or ASD recorded in the patient register during the study period, only 14% had ASD without an ID. Therefore, the effect of such misclassification should be minor.

Second, the national patient register does not contain information on visits to primary care. Therefore, the numbers presented in this study cannot be used to estimate the prevalence of pain among people with IDs. Moreover, in Sweden, specialist care, as a rule, requires a referral from primary care. If people with IDs differ in terms of chance of getting a referral to specialist care, this may bias the comparisons made between the ID and the gPop cohort.

Third, although the drug prescription register comprises data on all prescribed drugs dispensed at all pharmacies in Sweden, it does not contain information about over-the-counter-drugs or drugs provided to patients in inpatient care. However, among the drugs investigated in the present study, only paracetamol can be purchased without a prescription, and the interference of over-the-counter purchases should, therefore, be minor.

There are no biological or physiological reasons why patterns or the occurrence of pain would differ among people with IDs and those without IDs. Rather, we believe that the differences found between the two cohorts with respect to pain diagnoses are caused by other factors related to the individual, the caregivers, and the health care system. Diagnoses of pain are established through verbal communication between healthcare staff and the patient, physical examination, and laboratory tests (including radiology). It is, therefore, not surprising that, compared with their age peers in the general population, older people with IDs were less likely to get a diagnosis of headaches, musculoskeletal pain, or pain related to the circulatory and respiratory systems since they may not be able to report these symptoms. However, visceral pain and pain related to the urinary system are easier for the caregivers and healthcare staff to identify from bowel function, the appearance of urine, fever, local irritation, bacterial cultures, and other laboratory tests. This may explain why these types of pain are more common among people with IDs than in the general population.

Even in patients with full cognitive and physical function, diagnosing chronic pain is difficult. The recommendation is to assess patients with complex chronic pain in specialized pain rehabilitation teams consisting of pain physicians, physiotherapists, psychologists, and if needed, social workers and occupational therapists [40]. However, according to the clinical experience of the authors, people with IDs seldom have access to such teams and when they do, it is unlikely that team members with specialties other than developmental disorders will have sufficient knowledge and experience to provide care adapted to their special needs.

The pattern of differences in pain diagnoses between people with IDs and the general population was similar among men and women with the exception of visceral pain. In this study, having an ID was associated with an increased risk of diagnosis among men but not among women. This seemed to be driven by a low risk for visceral pain among women with IDs compared with women in the general population. There is a link between ovarian function and visceral pain [41,42]. Women with IDs are known to have earlier menopause than women in the general population. Therefore, even though the women in the two cohorts in the present study were of the same age (due to matching), the proportion of post-menopausal women was most likely higher in the ID cohort. Therefore, a possible explanation for the similarity in visceral pain diagnoses among older women with ID and the general population could be that one risk factor for such pain (i.e., being pre-menopausal) was less prevalent.

The recommended treatments for chronic pain are physiotherapy, Cognitive Behavioral Therapy (CBT), or Acceptance Commitment Therapy (ACT), either in individualized or in specialized pain rehabilitation programs [43,44]. Pharmacological treatment is also used but ideally as a “door-opener” to reduce pain symptoms [45]. Even so, the prescription of drugs for treating pain was common in both the ID and the gPop cohorts.

There was a pattern of increased prescription of paracetamol for people with IDs, regardless of pain diagnosis. In contrast, people with pain in the general population were more likely to have a prescription of a variety of pain treatments, such as COX(1+2) and COX2 inhibitors and weak opioids. There may be several reasons for this discrepancy among the two cohorts. People with IDs have an increased risk of polypharmacy compared to people without IDs [46]. Therefore, the choice to prescribe paracetamol may be based on a desire to not increase an already high number of drugs used by a person. The difference in the prescription of paracetamol could also be explained by a higher number of over-the-counter purchases in the general population than among people with IDs who may not have the same opportunity to visit pharmacies. Another potential explanation could be that there are financial considerations when prescribing paracetamol. In Sweden, prescribed drugs are subsidized by the state such that a single individual will never have to pay more than a set amount for prescribed drugs during a 12-month period. Therefore, it cannot be ruled out that physicians consider the lower economic standard of living among people with IDs and prescribe paracetamol rather than recommend over-the-counter purchases. However, the increased prescription of COX(1+2) and COX2 inhibitors as well as weak opioids may also reflect differences in healthcare access between the two cohorts. While paracetamol is often prescribed in primary health care, the drugs prescribed in the gPop cohort were most commonly done so by pain specialists rather than primary health care physicians. Therefore, the results may be an indication of better access to specialist care for people in the general population than people with IDs. Lastly, the fact that people in the general population were prescribed a greater range of drugs for pain treatment may be a reflection of a better follow-up of treatment effects and potential side effects in this group.

Even though only 1% of the people in the ID cohort were prescribed fentanyl during the study period, this was markedly more than in the general population. There is no “specific” pharmacological treatment for chronic pain, and unfortunately, strong opioids such as fentanyl are sometimes used as a last resort when nothing else helps. The problem with this is that the pain-relieving effect of strong opioids is temporary, while the cognitive and visceral side effects are ever present [47] and may well create a situation of general worsening of an ID patient’s symptoms of pain. Further studies should be performed to investigate if the prescription of fentanyl among older people with IDs truly is justified.

## 5. Conclusions

Compared with the general population, older people with IDs were more likely to be prescribed paracetamol and fentanyl and were less likely to be prescribed other drugs for pain. Whether this is due to physicians trying to avoid polypharmacy in a population that is known to have prescriptions to a multitude of drugs or if there are other reasons not to prescribe a greater range of pain treatments needs to be further investigated. However, it is important to closely evaluate all pharmacological treatments for pain among older patients with IDs as well as in all patient groups and to stop them if no significant effect can be recognized.

In Sweden, the use of fentanyl outside of operations and intensive care is mainly as plasters developed for cancer pain, but it is suspected that it has been overused in chronic, non-malignant cases during the last decade. This needs to be further investigated.

## Figures and Tables

**Table 1:** Number of people with different pain diagnoses in a group of 7936 older people (3609 women and 4327 men) with intellectual disabilities (ID) and a referent cohort from the general population (gPop), one-to-one matched by sex and year of birth.

Type of Pain	gPop			Intellectual Disabilities (ID)		
	Women	Men	Total	Women	Men	Total
	*n* (%)	*n* (%)	*n* (%)	*n* (%)	*n* (%)	*n* (%)
Headaches	106 (2.9)	71 (1.6)	177 (2.2)	53 (1.5)	42 (1.0)	95 (1.2)
Musculoskeletal pain	868 (24.1)	835 (19.3)	1703 (21.5)	537 (14.9)	480 (11.1)	1017 (12.8)
Pain related to the circulatory and respiratory systems	381 (10.6)	380 (8.8)	761 (9.6)	206 (5.7)	251 (5.8)	457 (5.8)
Visceral pain	422 (11.7)	292 (6.7)	714 (9.0)	407 (11.3)	454 (10.5)	861 (10.8)
Pain related to the urinary system	33 (0.9)	89 (2.1)	122 (1.5)	62 (1.7)	184 (4.3)	246 (3.1)

**Table 2:** Number of people with prescriptions of different pain medications in a group of 7936 older people (3609 women and 4327 men) with intellectual disabilities (IDs) and a referent cohort from the general population (gPop), one-to-one matched by sex and year of birth, stratified by pain diagnosis.

Type of Medication	gPop			ID		
	Women	Men	Total	Women	Men	Total
	*n* (%)	*n* (%)	*n* (%)	*n* (%)	*n* (%)	*n* (%)
**Headaches**						
COX(1+2) inhibitors	75 (71)	45 (63)	120 (68)	38 (72)	19 (45)	57 (60)
COX2 inhibitors	14 (13)	8 (11)	22 (12)	4 (8)	1 (2)	5 (5)
Paracetamol	62 (58)	45 (63)	107 (60)	44 (83)	25 (60)	69 (73)
Strong opioids	21 (20)	13 (18)	34 (19)	12 (23)	10 (24)	22 (23)
Weak opioids	58 (55)	45 (63)	103 (58)	26 (49)	22 (52)	48 (51)
Drugs for migraine	16 (15)	2 (3)	18 (10)	4 (8)	0 (0)	4 (4)
Antiepileptics	16 (15)	6 (8)	22 (12)	12 (23)	10 (24)	22 (23)
Tricyclic antidepressants	21 (20)	7 (10)	28 (16)	4 (8)	3 (7)	7 (7)
SNRI (Serotonin-norepinephrine reuptake inhibitor)	12 (11)	3 (4)	15 (8)	5 (9)	1 (2)	6 (6)
**Musculoskeletal Pain**						
COX(1+2) inhibitors	694 (80)	637 (76)	1331 (78)	350 (65)	295 (61)	645 (63)
COX2 inhibitors	128 (15)	90 (11)	218 (13)	26 (5)	13 (3)	39 (4)
Paracetamol	664 (76)	536 (64)	1200 (70)	460 (86)	382 (80)	842 (83)
Strong opioids	248 (29)	211 (25)	459 (27)	187 (35)	120 (25)	307 (30)
Weak opioids	548 (63)	482 (58)	1030 (60)	284 (53)	216 (45)	500 (49)
Drugs for migraine	47 (5)	14 (2)	61 (4)	12 (2)	0 (0)	12 (1)
Antiepileptics	100 (12)	59 (7)	159 (9)	60 (11)	62 (13)	122 (12)
Tricyclic antidepressants	99 (11)	39 (5)	138 (8)	25 (5)	13 (3)	38 (4)
SNRI	61 (7)	24 (3)	85 (5)	31 (6)	18 (4)	49 (5)
**Pain Related to the Circulatory and Respiratory Systems**						
COX(1+2) inhibitors	263 (69)	250 (66)	513 (67)	108 (52)	113 (45)	221 (48)
COX2 inhibitors	38 (10)	29 (8)	67 (9)	4 (2)	7 (3)	11 (2)
Paracetamol	229 (60)	210 (55)	439 (58)	152 (74)	165 (66)	317 (69)
Strong opioids	76 (20)	66 (17)	142 (19)	47 (23)	45 (18)	92 (20)
Weak opioids	207 (54)	186 (49)	393 (52)	93 (45)	83 (33)	176 (39)
Drugs for migraine	13 (3)	7 (2)	20 (3)	5 (2)	1 (0)	6 (1)
Antiepileptics	42 (11)	31 (8)	73 (10)	32 (16)	24 (10)	56 (12)
Tricyclic antidepressants	45 (12)	24 (6)	69 (9)	13 (6)	10 (4)	23 (5)
SNRI	23 (6)	14 (4)	37 (5)	13 (6)	13 (5)	26 (6)
**Visceral Pain**						
COX(1+2) inhibitors	314 (74)	182 (62)	496 (69)	213 (52)	178 (39)	391 (45)
COX2 inhibitors	47 (11)	18 (6)	65 (9)	8 (2)	7 (2)	15 (2)
Paracetamol	254 (60)	154 (53)	408 (57)	319 (78)	321 (71)	640 (74)
Strong opioids	93 (22)	60 (21)	153 (21)	104 (26)	84 (19)	188 (22)
Weak opioids	177 (42)	99 (34)	276 (39)	129 (32)	95 (21)	224 (26)
Drugs for migraine	20 (5)	5 (2)	25 (4)	10 (2)	0 (0)	10 (1)
Antiepileptics	55 (13)	23 (8)	78 (11)	57 (14)	52 (11)	109 (13)
Tricyclic antidepressants	57 (14)	14 (5)	71 (10)	20 (5)	15 (3)	35 (4)
SNRI	43 (10)	16 (5)	59 (8)	24 (6)	18 (4)	42 (5)
**Pain Related to the Urinary System**						
COX(1+2) inhibitors	18 (55)	54 (61)	72 (59)	27 (44)	61 (33)	88 (36)
COX2 inhibitors	5 (15)	2 (2)	7 (6)	5 (8)	3 (2)	8 (3)
Paracetamol	31 (94)	49 (55)	80 (66)	54 (87)	129 (70)	183 (74)
Strong opioids	16 (48)	21 (24)	37 (30)	21 (34)	53 (29)	74 (30)
Weak opioids	17 (52)	37 (42)	54 (44)	22 (35)	42 (23)	64 (26)
Drugs for migraine	0 (0)	1 (1)	1 (1)	1 (2)	1 (1)	2 (1)
Antiepileptics	10 (30)	11 (12)	21 (17)	4 (6)	20 (11)	24 (10)
Tricyclic antidepressants	5 (15)	9 (10)	14 (11)	5 (8)	8 (4)	13 (5)
SNRI	3 (9)	6 (7)	9 (7)	2 (3)	9 (5)	11 (4)

**Table 3:** Relative risks with 95% confidence intervals for the prescription of pain medication among 7936 older people with intellectual disabilities vs. a random sample from the general population, one-to-one matched by sex and year of birth, stratified by pain diagnosis.

Type of Pain	No Epilepsy	No Depression	
	Antiepileptics	Tricyclic Antidepressants	SNRI
Headaches	1.16 (0.56–2.40)	0.52 (0.22–1.22)	0.40 (0.09–1.76)
Musculoskeletal pain	0.90 (0.68–1.20)	0.44 (0.30–0.66)	0.80 (0.50–1.26)
Pain related to the circulatory and respiratory systems	0.92 (0.62–1.38)	0.44 (0.25–0.77)	0.99 (0.46–2.11)
Visceral pain	0.81 (0.58–1.13)	0.34 (0.22–0.53)	0.63 (0.37–1.05)
Pain related to the urinary system	0.37 (0.18–0.75)	0.47 (0.21–1.04)	0.70 (0.20–2.43)
